# Uncommon Cause of Chronic Vomiting in Children

**DOI:** 10.7759/cureus.25876

**Published:** 2022-06-12

**Authors:** Fatine El Aissaoui, Hanane Salhi, Abdelouhab Ammor, Houssain Benhaddou

**Affiliations:** 1 Department of Pediatric Surgery, Mohammed VI University Hospital, Oujda, MAR

**Keywords:** pediatrics, heineke-mikulicz, non- bilious vomiting, pyloroplasty, pyloric stenosis

## Abstract

Gastric outlet obstruction (GOO) is unusual and must be suspected in children with chronic vomiting and abnormal weight status. The treatment depends on etiology, and surgery is not always the first remedy. Diagnosis is easily confirmed by upper gastrointestinal fibroscopy.We report the case of an 11-year-old girl, who was presented with non-bilious emesis and weight loss. Abdominal computed tomography, ultrasound, and upper gastrointestinal fibroscopy showed dilated stomach with pyloric stenosis, which was confirmed by abdominal laparoscopic exploration and cured by Heineke-Mikulicz pyloroplasty. We also compare our study to previously reported cases.

## Introduction

Gastric outlet obstruction (GOO) is rare in children, except in cases due to infantile hypertrophic pyloric stenosis. Primary acquired GOO has been seldom reported and consequently remains poorly understood. Moore has classified congenital obstructions in 1986, including antral and pyloric atresia and webs [1]. In 1997, the classification was completed by Sharma et al., with the addition of primary acquired obstructions [2]. We describe the case of an 11-year-old girl with GOO, who was treated with Heineke-Mikulicz pyloroplasty. The objective of this case report is to discuss and compare our attitude toward different therapeutic modalities previously reported in the literature.

## Case presentation

**Figure 1:**
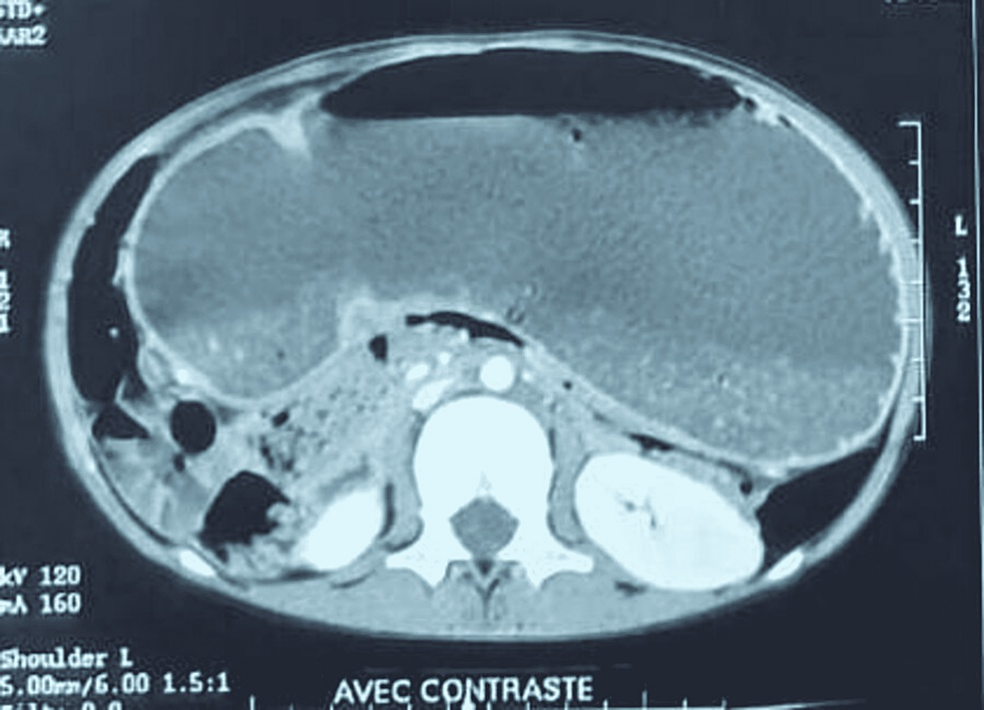
Abdominal computed tomography showing dilated stomach

**Figure 2:**
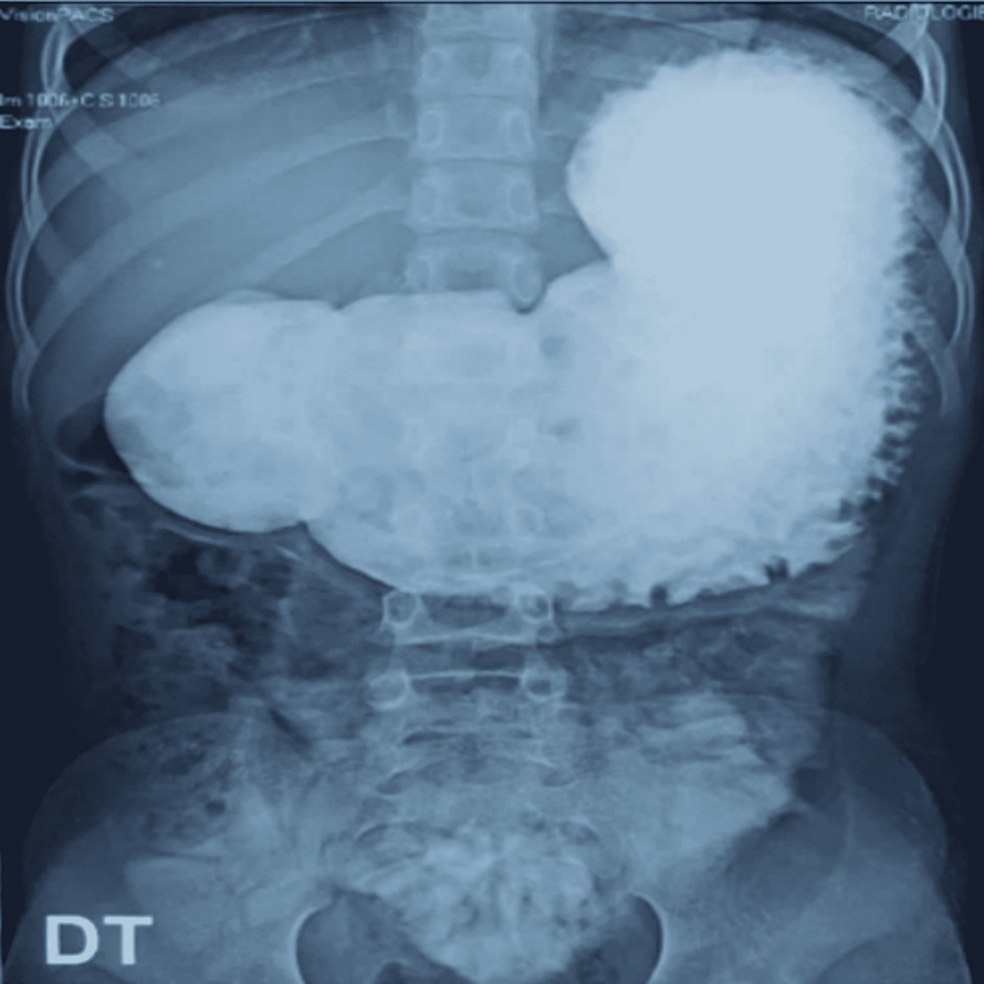
Upper gastrointestinal radiogram showing stomach dilatation

**Figure 3:**
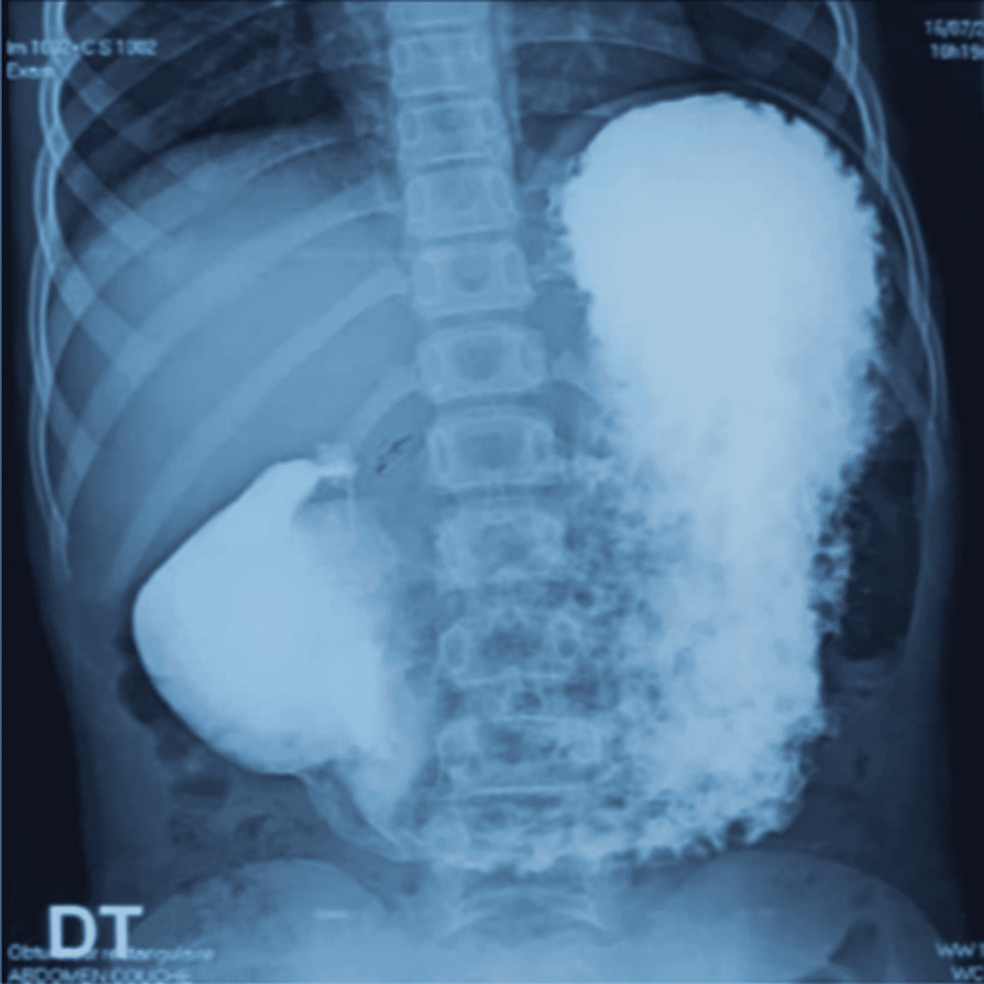
Upper gastrointestinal radiogram showing pyloric incomplete stenosis with normal duodenum opacification

**Figure 4:**
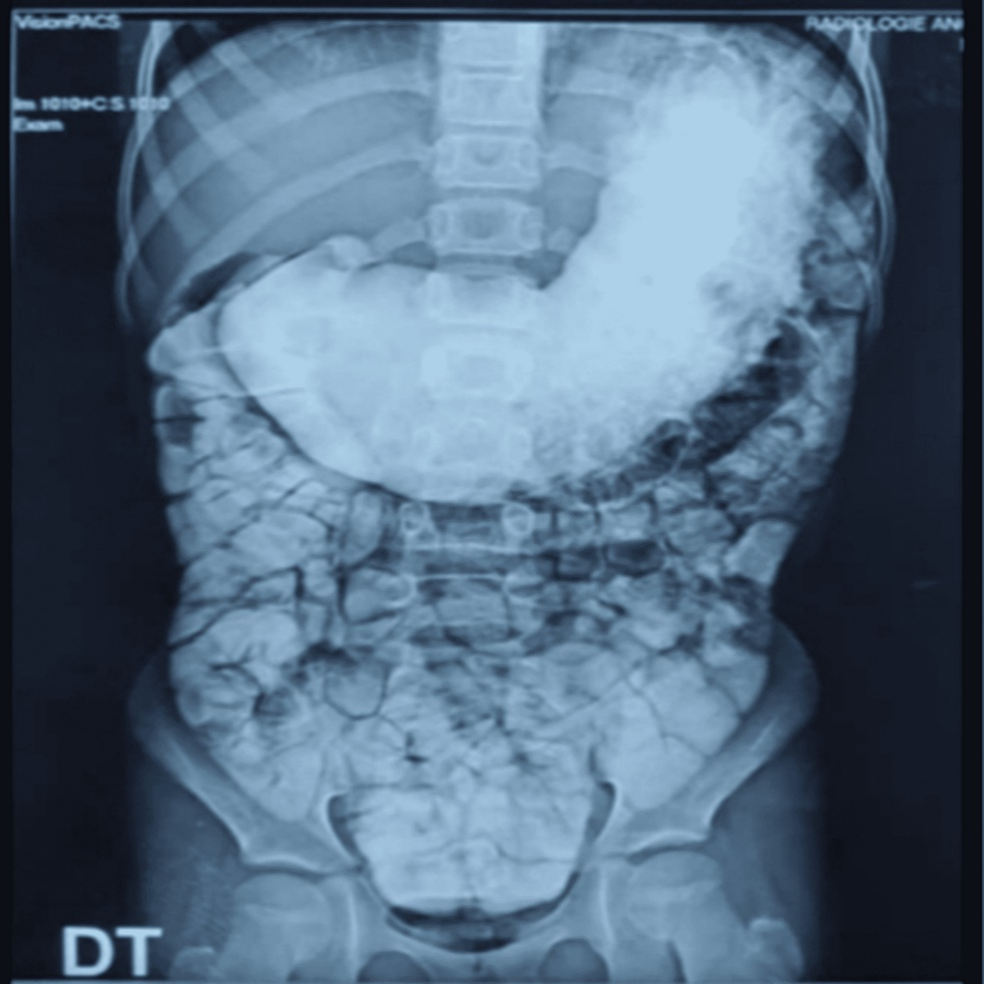
Upper gastrointestinal radiogram showing distended stomach with complete downstream digestive opacification

An 11-year-old girl was admitted to the pediatric surgery department, with non-bilious postprandial vomiting after every meal for the past four months, without transit disorder. The patient had no past surgical interventions. The clinical examination showed an apyretic child with failure to thrive (-2 SD for weight and -1 SD for height). The abdominal examination was normal, and there were no other coexisting problems. A biological checkup revealed anemia (hemoglobin: 8.8 g/dL) and hypoprotidemia (53 g/L). Abdominal computed tomography was performed, which showed a significant gastric stasis upstream a duodenal stenosis (Figure 1). Abdominal ultrasound showed distended stomach without signs of pyloric hypertrophy. Upper digestive fibroscopy revealed a dilated stomach, fundic petechial gastritis, and a passable pyloro-bulbar stenosis. Duodenum was normal. Interstitial gastritis was confirmed by pathology of gastric and duodenal biopsies. Testing of *Helicobacter pylori *infection was negative. Upper GI revealed an important gastric stasis and an incomplete pyloro-duodenal stenosis with a normal duodeno-jejuno-ileal flow (Figures 2-4).

The therapeutic attitude for our patient was Heineke-Mikulicz pyloroplasty. The abdomen was explored by means of laparoscopy. The stomach was very dilated with apparent pyloric stenosis, but without muscle hypertrophy. There was no external compression from the surroundings. Postoperative follow-up was uneventful. The patient started a progressive replenishment. The evolution was favorable after a follow-up period of two years, and the patient progressed smoothly on to a normal diet, gained weight, and the emesis disappeared.

## Discussion

In 1997, Sharma et al. described five cases of pyloric stenoses, in which the symptomatology was similar to ours [2]. Since then, several researchers have described its occurrence [2]. Many terms have been used to describe this disease, including acquired GOO during infancy and childhood [2], primary acquired GOO [2-3], late-onset primary acquired GOO [4], non-obstructive delayed gastric emptying in children [5], pyloric achalasia [6], and non-hypertrophic pyloric stenosis [7].

Around 40-50% of pyloric atresia have associated anomalies (esophageal, gastrointestinal, renal, or biliary disease) [8]. Epidermolysis bullosa is a particular association, characterized by epithelial weakness, leading to blisters formation and skin erosions by dermo-epidermal junction cleaving [9]. In this case, a local injury appears in pyloric mucosa with bad retractile healing, which causes obstruction [10]. This association is called Herlitz syndrome, which is nearly always lethal with severe infections, contributing to sepsis and serious hydro-eletrolytric disorders [10]. On the one hand, prognosis is excellent once the GOO is isolated, but on the other hand, it is life threatening as a consequence of epidermolysis bullosa association.

If the peptic origin cannot be clearly ruled out, other etiologies have to be considered. Congenital disease is reported in about 1 per 100,000 newborns, including pyloric atresia, and pyloric and antric webs. In case of complete obstruction, antenatal diagnosis is possible, and abdominal ultrasound shows a voluminous stomach and hydramnios. The symptoms appear during the neonatal period, with non-bilious emesis [8]. Concerning incomplete obstructions, especially antric webs, the signs are belatedly predicted to occur [11]. Upper gastrointestinal and fibroscopy confirmed the diagnosis most of the time.

Surgery is rare in pyloric stenosis, in which the etiology is peptic, and can be proposed after several weeks of medical treatment. However, when the stenosis is congenital and tardily revealed, or acquired GOO is idiopathic, the surgery has to be suggested straight away. Heineke-Mikulicz pyloroplasty remains the standard curative treatment in most reported cases, which was the main treatment for our patient, and this procedure can now be performed laparoscopically [12].

In 2006, Hameury et al. found that the pneumatic dilatation was ineffective and that the cure was completed by surgery [7]. In 2007, Lin et al. reported the treatment of two cases with pneumatic dilatation of the pylorus instead of Heineke-Mikulicz pyloroplasty [6]. In 2008, Sharma et al. stated that pneumatic dilatation of the pylorus might be the best treatment modality for primary acquired GOO in infancy and childhood [13]. Later, in 2010, Karnsakul et al. cured an 18-month-old boy with non-peptic, non-hypertrophic pyloric stenosis after three sessions of endoscopic pyloric balloon dilatation [14]. Jawaid et al. found no difference in regard to symptomatic improvement after pneumatic dilatation [5]. Some studies reported that permanent gastric electrical stimulation may also represent a successful therapy option for children and adolescents with primary acquired GOO. For our patient, we did not try the pneumatic dilatation. He received symptomatic treatment at first, but without successful results, and then the surgery was performed.

We compare our study to a similar case reported by Härter et al. in 2017 of a five-year-old boy with non-bilious vomiting and a slightly reduced nutritional status [15]. In the ultrasound examination, the stomach was well-filled after ingestion of water. Only a small volume of fluid passed over the pylorus, without distinguishable obstacle. Upper gastrointestinal endoscopy revealed normal findings, unlike our case, which revealed dilated stomach with gastritis. Upper gastrointestinal series showed pronounced pyloric stenosis, with delayed passage of contrast medium into the duodenum. Contrary to our therapeutic attitude, they began with a trial therapy, and endoscopic botulinum toxin injection was administered. Vomiting and abdominal pain ceased for seven days but then recurred. Hence, Heineke-Mikulicz pyloroplasty was performed. Upper gastrointestinal series carried out at one month postoperatively showed timely passage of contrast medium from the stomach to the duodenum, without any evidence of an obstacle.

Other causes of GOO are classified by Moore as follows: antral gap atresia, pyloric gap atresia, pyloric septum, pyloric membrane, antral membrane, or web [1]. Treatment of congenital GOO due to pyloric atresia or gastric antral webs should be surgical. Gap atresia is cured by gastroduodenostomy or gastrostomy. For antral membrane, excision of the membrane is chosen only when it is more than 1cm from the pylorus, but when it is 1cm or less, excision of the membrane and Heineke-Mikulicz pyloroplasty should be chosen. For pyloric membrane, excision of the membrane and Heineke-Mikulicz pyloroplasty are recommended, and for solid pyloric atresia, the treatment consists of excision and gastroduodenostomy.

## Conclusions

Pediatric GOOs are uncommon. Their causes and treatments are debated. Non-bilious vomiting with weight loss must be explored in children out of the usual age of hypertrophic pyloric stenosis. Fibroscopy and upper gastrointestinal are essential to approve the diagnosis. In case of medical treatment failure, the pyloroplasty allows most often to solve the problem.
